# Rapid hyperpolarization and purification of the metabolite fumarate in aqueous solution

**DOI:** 10.1073/pnas.2025383118

**Published:** 2021-03-22

**Authors:** Stephan Knecht, John W. Blanchard, Danila Barskiy, Eleonora Cavallari, Laurynas Dagys, Erik Van Dyke, Maksim Tsukanov, Bea Bliemel, Kerstin Münnemann, Silvio Aime, Francesca Reineri, Malcolm H. Levitt, Gerd Buntkowsky, Alexander Pines, Peter Blümler, Dmitry Budker, James Eills

**Affiliations:** ^a^Eduard-Zintl-Institute for Inorganic Chemistry and Physical Chemistry, Technical University Darmstadt, 64287 Darmstadt, Germany;; ^b^GSI Helmholtzzentrum für Schwerionenforschung GmbH, Helmholtz-Institut Mainz, 55128 Mainz, Germany;; ^c^Department of Chemistry, University of California, Berkeley, CA 94720;; ^d^Department of Molecular Biotechnology and Health Sciences, Center of Molecular Imaging, University of Turin, 10126 Turin, Italy;; ^e^Department of Chemistry, University of Southampton, Southampton, SO17 1BJ, United Kingdom;; ^f^Department of Mechanical and Process Engineering, Technical University of Kaiserslautern, 67663 Kaiserslautern, Germany;; ^g^Institute for Physics, Johannes Gutenberg University, D-55090 Mainz, Germany

**Keywords:** hyperpolarization, parahydrogen, metabolism, MRI, biomarker

## Abstract

Hyperpolarized fumarate is a promising biosensor for carbon-13 magnetic resonance metabolic imaging. Such molecular imaging applications require nuclear hyperpolarization to attain sufficient signal strength. Dissolution dynamic nuclear polarization is the current state-of-the-art methodology for hyperpolarizing fumarate, but this is expensive and relatively slow. Alternatively, this important biomolecule can be hyperpolarized in a cheap and convenient manner using parahydrogen-induced polarization. However, this process requires a chemical reaction, and the resulting solutions are contaminated with the catalyst, unreacted reagents, and reaction side-product molecules, and are hence unsuitable for use in vivo. In this work we show that the hyperpolarized fumarate can be purified from these contaminants by acid precipitation as a pure solid, and later redissolved to a desired concentration in a clean aqueous solvent. Significant advances in the reaction conditions and reactor equipment allow for formation of hyperpolarized fumarate at ^13^C polarization levels of 30–45%.

## Significance

Magnetic resonance imaging is hindered by inherently low sensitivity, which limits the method for the most part to observing water molecules in the body. Hyperpolarized molecules exhibit strongly enhanced MRI signals which opens the door for imaging low-concentration species in vivo. Biomolecules can be hyperpolarized and injected into a patient allowing for metabolism to be tracked in real time, greatly expanding the information available to the radiologist. Parahydrogen-induced polarization (PHIP) is a hyperpolarization method renowned for its low cost and accessibility, but is generally limited by low polarization levels, modest molecular concentrations, and contamination by polarization reagents. In this work we overcome these drawbacks in the production of PHIP-polarized [1-^13^C]fumarate, a biomarker of cell necrosis in metabolic ^13^C MRI.

NMR spectroscopy and imaging are analytical techniques used to noninvasively extract information about the structure or composition of a sample. Unfortunately, these methods are not always sufficiently sensitive, and in order to overcome the limitations this imposes, hyperpolarization methods have been developed to produce samples with enhanced magnetic resonance signals. Signal enhancements on the order of 10^5^ can be achieved for solution-state samples using hyperpolarization techniques such as dissolution dynamic nuclear polarization (dDNP) (1, 2) or parahydrogen-induced polarization (PHIP) (3–5). This allows the injection of hyperpolarized probe molecules in vivo, and subsequent imaging of metabolism (6–10). One such example is the imaging of hyperpolarized fumarate, which is converted to malate in one step of the Krebs cycle, and acts as a sensitive probe of cell necrosis (11–18). This has been demonstrated in preclinical studies as, for example, a method to image tumor response to therapy (11) and acute kidney injury (15) or myocardial infarction (17).

For preclinical studies the currently preferred method to hyperpolarize fumarate is dDNP, but polarization build-up times on the order of an hour are typical, and the expense and high technical demands of this method strongly limit its widespread application (19). It was recently shown that hyperpolarized fumarate can be formed via PHIP (20, 21), which is advantageous since it is much easier to implement, and 1–2 orders of magnitude less expensive than dDNP (2, 4). A molecular precursor is chemically reacted with hydrogen gas enriched in the *para-*nuclear spin isomer, and this is followed by a magnetic field cycle (22, 23) to transfer the *para-*spin order from the hydrogen nuclei to the ^13^C nucleus in fumarate. It is necessary to polarize the ^13^C nucleus for in vivo imaging because the hyperpolarized ^13^C nuclei typically relax back to thermal equilibrium slower than ^1^H nuclei, and have a large chemical shift dispersion which allows chemical selectivity. The drawback to producing hyperpolarized fumarate via PHIP is that, in addition to the desired fumarate product, the reaction solutions contain a plethora of additional chemicals, most notably the ruthenium catalyst, unreacted reagents, and reaction side products.

In this work we demonstrate that parahydrogen-polarized fumarate solutions can be purified of contaminants by acid precipitation of the fumarate, and subsequent redissolution of the pure material in a clean aqueous solvent. This physicochemical manipulation is possible because the solubility of fumarate is significantly reduced in acidic solution (24). We perform the hydrogenation reaction in a steel reactor, which allows us to work with high hydrogen flow rates, and hence form hyperpolarized fumarate at higher concentrations and volumes than was demonstrated in previous work (21). This makes PHIP competitive with dDNP for producing nuclear spin-polarized fumarate, since we show that it can be formed in higher concentrations and with higher ^13^C polarization.

Signal enhancement or polarization level alone does not indicate the total signal intensity available from the hyperpolarized species, which for many applications is a crucial parameter. We adopt molar polarization as a key figure of merit, which we take as the product of the carbon-13 polarization times the concentration of [1-^13^C]fumarate molecules. For in vitro or in vivo studies using hyperpolarized biomolecules, important criteria for the solutions prior to injection/perfusion are (25): 1) concentration of the hyperpolarized species; 2) polarization level (usually >10% is desirable); 3) purity from toxic contaminants, and; 4) biocompatibility of the solution, i.e., being at physiological pH, temperature, and osmolarity. In this paper we will address these points in the context of our work forming fumarate via PHIP.

## Results

**Fig. 1.**
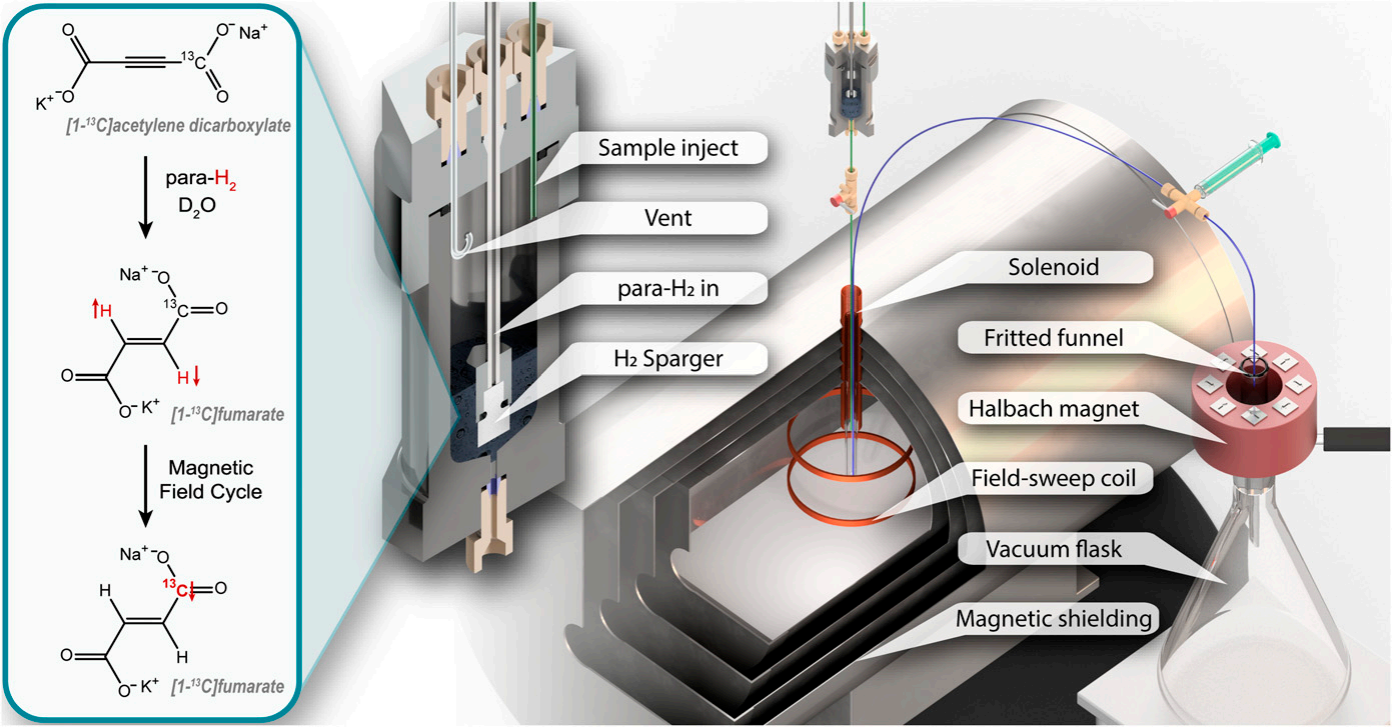
The experimental apparatus used in this work. An expansion of the reactor is shown for clarity. On the left the chemical reaction and magnetic field cycle step are shown, with red arrows and atom labels representing the hyperpolarized nuclei.

To produce hyperpolarized fumarate, *para*-enriched hydrogen gas was rapidly bubbled through a precursor solution (*Materials and Methods*) in a heated steel reactor. The sample was then ejected into a magnetically shielded chamber, and a magnetic field cycle was used to transfer the ^1^H singlet order in fumarate (originating from the parahydrogen protons) into ^13^C magnetization of the carboxylate carbon. In experiments involving a purification step, part of this solution was mixed with a concentrated sodium fumarate solution (to raise the overall fumarate concentration), and then mixed with HCl which lowered the pH and caused fumaric acid to precipitate out of solution. The residual reaction solution was filtered off and the fumaric acid crystals were redissolved in aqueous solution. The precipitation and redissolution steps were carried out in a 100-mT Halbach permanent magnet array to preserve the hyperpolarized spin order throughout. Unless otherwise stated, experiments were performed without isotopic enrichment of the precursor molecule, and the ∼2.2% of molecules containing a ^13^C spin in the carboxylate position were observed in the NMR experiments. A schematic showing the chemical reaction and experimental apparatus is shown in Fig. 1.

### Optimizing Molar Polarization.

**Fig. 2.**
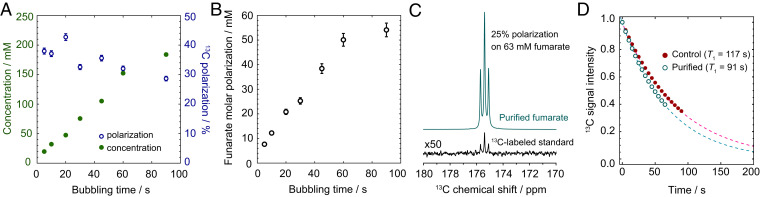
(*A*) The polarization and concentration of fumarate formed for different durations of parahydrogen bubbling, under the experimental conditions described in the text. (*B*) Molar polarization of [1-^13^C]fumarate, i.e., the product of concentration and polarization. We do not account for non–^13^C-enriched material being used in these experiments, so the true values are ∼45× lower. (*C*) The ^13^C NMR spectra of a purified hyperpolarized fumarate solution (at natural ^13^C abundance) and a standard solution of 500 mM [1-^13^C]fumarate with thermal equilibrium spin polarization. Both spectra were acquired using one transient, and are shown with 0.3-Hz line broadening. (*D*) ^13^C $T_{1}$ data for the control and purified samples. Each data point shows the integral of the [1-^13^C]fumarate resonance in the corresponding spectrum. The ^13^C signal intensity in both datasets is normalized to 1 for the first data point, and the dotted lines are monoexponential decays of the form ${\text{e}}^{-t/T_{1}-t/T_{\text{P}}}$, fit to the data using the stated $T_{1}$ values and $T_{\text{P}}$ = 327 s to account for magnetization lost due to successive pulses as described in the text.

Since it is important to produce hyperpolarized fumarate in high concentration and with high ^13^C polarization, we performed the chemical reaction at 8.5 bar with varied bubbling duration to find an optimum. In each experiment, after the chemical reaction and magnetic field cycle, the ^13^C-polarized fumarate solution was transferred to a benchtop NMR spectrometer for ^13^C signal acquisition. After the hyperpolarized NMR signals had relaxed, a thermal equilibrium ^1^H NMR spectrum was acquired on each sample to quantify the fumarate concentration. The results of these experiments are shown in Fig. 2*A*, and further experimental details are provided in *Materials and Methods*. The concentration of fumarate increases approximately linearly with bubbling duration up to 150 mM after 60 s, but the fumarate formation slows beyond this, and 180 mM is produced after 90 s. The polarization decreases as bubbling duration is increased due to nuclear spin relaxation of the fumarate ^1^H singlet state.

In Fig. 2*B* we show the molar polarization that would arise if [1-^13^C]-labeled material were used, which effectively scales the molar polarization by a factor of ∼45 relative to the unlabeled case. This relies on the assumption that the reaction and polarization transfer efficiency does not depend on the abundance of ^13^C.

### Purification of Hyperpolarized Fumarate.

To test the precipitation/redissolution purification method, the same reaction and field-cycle procedure was used as before, with a 60-s bubbling duration chosen as it led to formation of a high concentration of fumarate (150 mM) at a relatively high polarization level. After performing the reaction and magnetic field cycle, the ^13^C-polarized fumarate solution was split into a control sample and a sample that was purified. The purified solution was transported to the benchtop NMR spectrometer for signal acquisition following a π/2 pulse. Immediately following this, the control sample was measured in the same manner. For both samples, after the hyperpolarized NMR signals had fully relaxed, a thermal-equilibrium ^1^H NMR spectrum was acquired to quantify the fumarate concentration. The time between sample ejection from the reactor and detecting ^13^C spectra of the purified and control samples was ∼30 and 34 s, respectively. Further experimental details are provided in *Materials and Methods*.

To quantify the polarization level we compare a ^13^C spectrum of a hyperpolarized sample that underwent the purification procedure, and a thermal equilibrium ^13^C spectrum of a 500 mM isotopically-enriched [1-^13^C]fumarate calibration sample. Both spectra were acquired using a π/2 pulse and the results are shown in Fig. 2*C*. The concentration of hyperpolarized fumarate was 100–150 mM, which was insufficient for the desired rapid precipitation. We therefore added an aliquot of highly concentrated fumarate solution to raise the overall concentration to ∼350 mM, which allowed for the precipitation to occur on a 1-s timescale. We calculate that the polarization of the hyperpolarized fumarate molecules (63 mM) in the purified sample (after addition of the unpolarized aliquot) was 25%. The addition of unpolarized fumarate is not necessary for the purification step but speeds up the rate of precipitation which is convenient for this preliminary demonstration.

To compare the ^13^C $T_{1}$ times of the purified and control samples, a modified version of the experiment was used, where instead of applying a 90° flip-angle pulse, 10° pulses were used to excite the ^13^C signals for acquisition once every 5 s. By using a small flip-angle pulse the hyperpolarization was not significantly perturbed for each signal acquisition, and the longitudinal relaxation time ($T_{1}$) of the carboxylate ^13^C spins could be measured from the signal decay in a single experiment. The effective relaxation ($T_{\text{P}}$) induced by the pulses corresponds to $T_{\text{P}}$ = 327 s, which is explained in the *SI Appendix*. The results of this experiment are shown in Fig. 2*D*. The ^13^C $T_{1}$ was measured to be 117 s for the control sample, and 91 s for the purified sample. This difference is likely due to the samples containing different concentrations of paramagnetic oxygen; the control sample was thoroughly oxygen degassed during the hydrogenation reaction, but the aqueous solvent used to redissolve the purified fumarate was not.

**Fig. 3.**
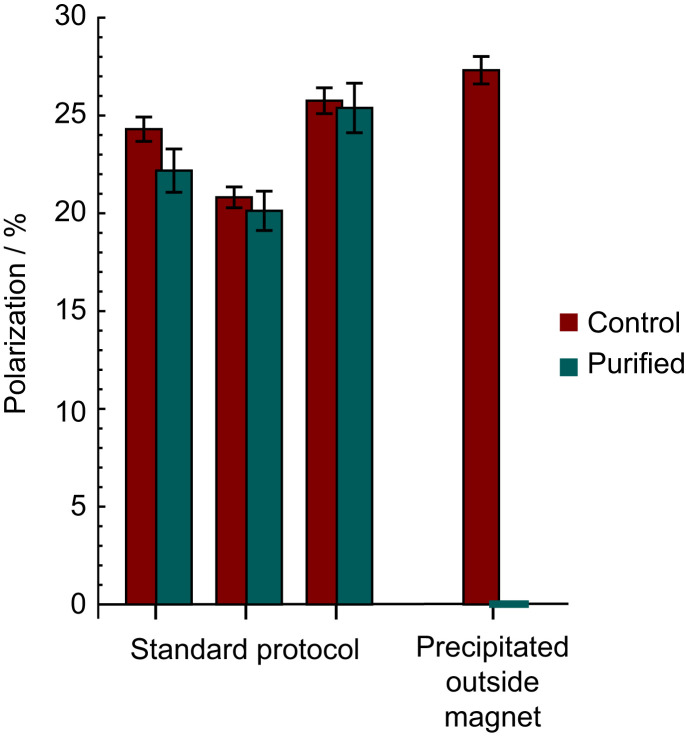
The polarization of the hyperpolarized [1-^13^C]fumarate molecules for the experiments described in the text. The shown error bars are dominated by volume uncertainty of the syringe measurements.

As specified before, in vivo applications require high-purity material. However, due to finite spin-relaxation times it is important that the purification step is rapid, and that there is no significant polarization loss during the purification procedure. Such loss may in principle also result during the phase transition. The experiment was performed, and we compared the ^13^C polarization level of the control sample to the purified sample. To verify the reproducibility, the experiment was repeated three times; the results are shown in Fig. 3. Within the given errors there is no evidence that the phase transitions have any effect on nuclear spin polarization. Note that the polarization level values in Fig. 3 are generally lower than in Fig. 2*A*; this is the result of additional relaxation occurring in the ∼30 s of the precipitation procedure.

For completeness, one experiment was performed in which the purification procedure was performed outside the magnet, and, as expected, the ^13^C polarization was lost. The loss of nuclear magnetization when precipitating the sample in a low magnetic field is not yet fully understood. It could be associated with rapid spin-relaxation processes in low magnetic field for the small suspended particles involved in the nucleation process, or efficient contact between the Zeeman and dipolar orders in the solid state at low magnetic field.

### Fumarate Metabolic Reaction.

**Fig. 4.**
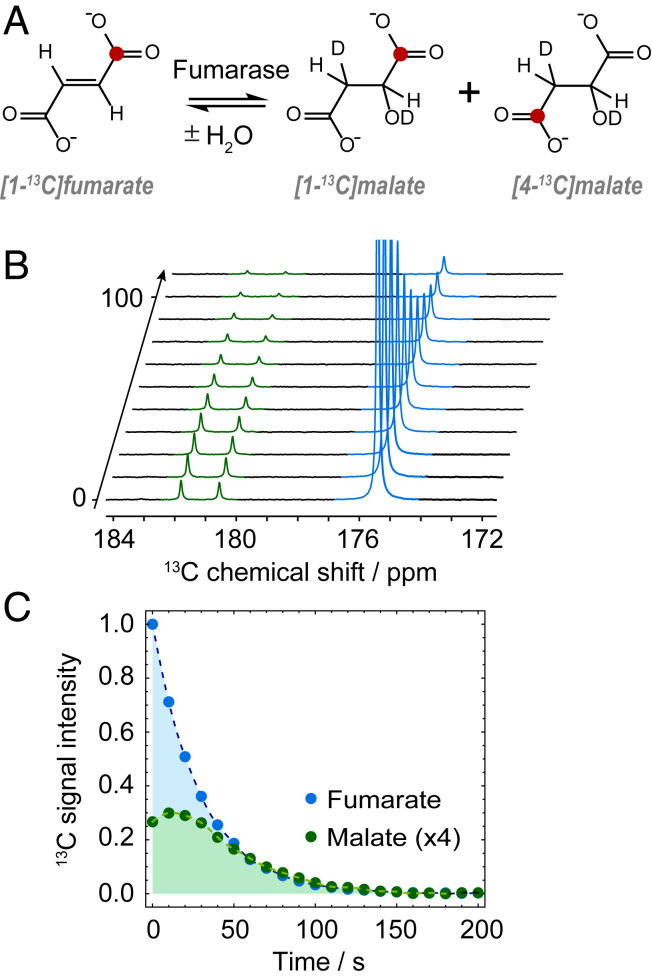
(*A*) The enzyme-catalyzed metabolism of fumarate into malate, with ^13^C labels shown in red. (*B*) A series of ^13^C NMR spectra of a purified hyperpolarized fumarate solution after addition to a phosphate buffer containing fumarase enzyme, showing metabolism of fumarate to malate. (*C*) Integrals of the fumarate and malate ^13^C NMR signals normalized to 1 for the first fumarate signal, with the malate signals multiplied by 4 for clarity.

A sample of hyperpolarized fumarate was generated as previously described, but with 20% [1-^13^C] isotopic enrichment of the starting material. After sample precipitation, the fumarate was redissolved in a phosphate buffer, and the resulting solution was mixed with fumarase enzyme. The ^13^C NMR signals were acquired every 7 s using 15° flip-angle pulses to monitor the enzyme-catalyzed conversion of fumarate to malate, and the results are shown in Fig. 4. Further experimental details are given in *Materials and Methods*. The hyperpolarized [1-^13^C]fumarate signal at 175.4 ppm can be seen to decay, and two additional resonances corresponding to [1-^13^C]malate and [4-^13^C]malate appear at 181.8 and 180.6 ppm, respectively. At first the malate signals grow in intensity as the initial rate of metabolism is high, and then decay predominantly due to nuclear spin relaxation.

### Quantifying Contamination.

To quantify how effectively the precipitation/redissolution procedure excluded the catalyst molecules from the solutions, inductively coupled plasma–optical emission spectrometry (ICP-OES) elemental analysis was performed on a purified sample. The standard procedure was used to purify the fumarate, but with an additional washing step; while on the sinter, the solid fumarate powder was washed twice with deionized water, and twice with acetone to remove residual catalyst solution from the surface of the crystals. The catalyst concentration in the final solution was determined by ICP-OES to be 16 µM, which corresponds to a reduction from the initial concentration of more than 99.7%.

**Fig. 5.**
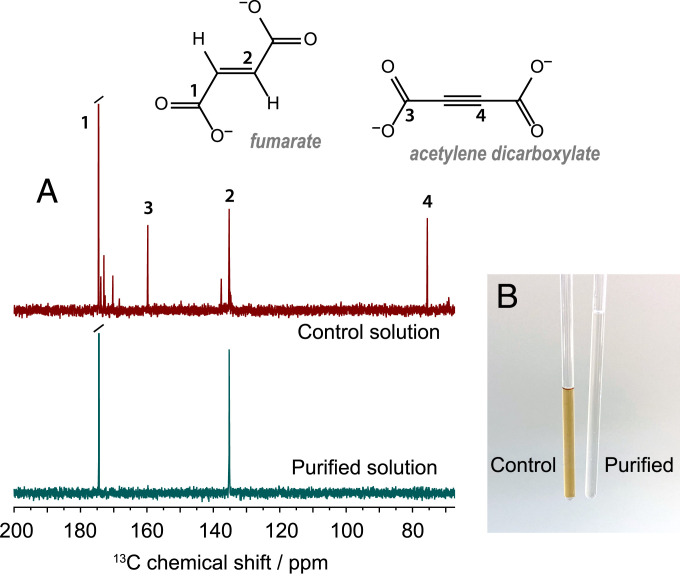
(*A*) A comparison between thermal equilibrium ^13^C NMR spectra for a purified and a control sample, acquired in an 11.7-T magnet. In the control sample spectrum some known resonances are labeled, with additional resonances likely present due to reaction side-products. (*B*) A photograph of control and purified samples in 5-mm NMR tubes.

Thermal equilibrium ^13^C NMR spectra were acquired of the control and purified solutions in an 11.7-T magnet. The 1,024-scan spectra were acquired with ^1^H decoupling, using a prescan delay of 30 s and 30° flip-angle pulses. The results are shown in Fig. 5, and qualitatively show contamination of the unpurified solution with the acetylene dicarboxylate starting material and unassigned reaction side-products, but no contaminants are visible in the purified sample spectrum. From these spectra, we can set an upper bound on the concentration of starting material in the purified solution at 0.6 mM, which corresponds to less than 0.4% of the fumarate concentration. Thermal equilibrium ^1^H NMR spectra of the control and purified samples are included in *SI Appendix*, which show contaminant molecules at a level of less than 1% compared to the fumarate concentration. Additionally, high-performance liquid chromatography (HPLC) spectra were acquired of the control and purified samples, and these data are included in *SI Appendix*.

### Precipitation without Addition of Unpolarized Fumarate.

For the precipitation experiments described, the reaction was run at 8.5-bar parahydrogen pressure, and ∼150 mM fumarate was produced after 60 s. Immediately before the precipitation step the hyperpolarized reaction sample was mixed with a 1 M sodium fumarate solution to raise the overall fumarate concentration, and hence speed up the rate of precipitation. The parahydrogen generator limited the maximum pressure in these experiments to 10 bar, and it was determined that 8.5 bar was suitable from a safety perspective. However, for a proof-of-principle demonstration an experiment was performed using 20-bar hydrogen gas (not *para*-enriched) and 500 mM of starting material (instead of 250 mM), with all other reaction conditions held constant. During the 60-s bubbling period 240 mM fumarate was produced, and solid fumarate was precipitated directly from this solution in <2 s. Although these experiments were not performed using *para*-enriched hydrogen gas, we have no reason to believe the ^13^C polarization levels would be different when precipitating directly from the reaction solution.

## Discussion

We adopt the unit molar polarization, which is a useful measure of the signal one might expect from a hyperpolarized sample in a fixed-volume detector. This is more comprehensive than reporting signal enhancement or polarization alone, which can in many hyperpolarization experiments be improved by using lower and lower substrate concentrations (26), without increasing the observable NMR signal. We report 50 mM molar polarization (assuming [1-^13^C] isotopic enrichment of the starting material) from the control samples which used a 60-s hydrogenation reaction. For the purification step the hyperpolarized reaction samples were mixed with a 1 M sodium fumarate solution to raise the overall fumarate concentration, and speed up the rate of precipitation. This diluted the concentration of hyperpolarized fumarate molecules, and hence reduced the molar polarization to 16 mM. We showed in subsequent experiments that by using higher hydrogen gas pressure and starting material concentration, fumarate can be formed at high enough concentration to be precipitated directly from the reaction solution. The rate of precipitation is nonlinear with fumarate concentration, and we have qualitatively observed that above ∼200 mM the precipitation is rapid. Additional experimental steps such as performing the precipitation at lower temperature and optimizing the volume of acid added should improve the rate and efficacy of the precipitation.

The residual ruthenium catalyst concentration in a 1 mL purified fumarate solution was 16 µM. For comparison, the phase extraction used for preclinical experiments with PHIP-polarized pyruvate produces solutions contaminated with ∼30 μM residual rhodium catalyst (9). The toxicity of other ruthenium(II) complexes has been investigated in mice and the LD_50_ was found to be >2,000 mg kg^−1^ (27), and the IC_50_ in human nontumor breast cells was found to be 1–20 μM (28).* These results indicate that the purity of our solutions from the catalyst should allow for in vitro and preclinical studies since the bolus of hyperpolarized solution will be further diluted in the blood. It will be necessary to investigate the toxicity of the catalyst, precursor, and reaction side-product molecules, which are currently unknown. The purification procedure implemented in this work involved only a single step; more thorough rinsing or the addition of a second precipitation/dissolution step would likely reduce the degree of contamination further. This is the subject of ongoing research.

Catalyst scavenging has been demonstrated as a way to purify hyperpolarized solutions of the catalyst molecules (29, 30). Using physicochemical manipulations to purify the target molecule from solution as demonstrated here has a number of notable advantages: 1) it is in principle a much faster chemical process, meaning less polarization is lost due to relaxation; 2) the target molecule can be purified of all contaminants, rather than only the catalyst molecules; 3) the dissolution liquid can be chosen to yield a final solution at physiological conditions; and 4) the pure material can be redissolved at an arbitrary concentration, up to its solubility limit, which can lead to significantly higher molar polarization in the final solution. The volume scale for the experiments in this work was a few milliliters, and we expect that this could be scaled up by an order of magnitude with relative ease. The limiting factor would likely be the rate at which parahydrogen gas could be supplied; here we hydrogenated 2 mL of precursor solution by flowing hydrogen gas at 6 L/min for 1 min at either 8.5 or 20 bar.

In this work we achieve ^13^C polarization levels higher than those reported for dDNP experiments, although we note that dDNP could produce fumarate solutions with higher molar polarization since the [1,4-^13^C_2_]-isotopomer can be used with both carbons in principle polarized up to 100%. When fumarate is formed via reaction with parahydrogen, the proton singlet state can be converted into 100% polarization of the ^13^C spin in the [1-^13^C] isotopomer (an AA′X spin system), or 50% polarization of each ^13^C spin in the [1,4-^13^C_2_] isotopomer (an AA′XX′ spin system) (31).

In conclusion, we have demonstrated that hyperpolarized [1-^13^C]fumarate can be formed in concentrations of more than 100 mM via PHIP, with typical ^13^C polarization levels of 30–45%. We have shown that acid precipitation as a pure solid and subsequent redissolution as a salt is an effective method to purify the hyperpolarized [1-^13^C]fumarate from toxic contaminants in the chemical reaction solution, and that there is no measurable polarization loss caused by the precipitation and redissolution to within the measurement error. The method presented here was demonstrated on fumarate because it rapidly precipitates in acidic solution; we anticipate that exploiting the physicochemical properties of other molecules will allow for the purification of alternative hyperpolarized targets employing precipitation techniques. Since the ^13^C $T_{1}$ in the solid state significantly exceeds (24) the solution-state ^13^C $T_{1}$, systematic studies of the hyperpolarization lifetime in the precipitated state are an exciting future prospect.

## Materials and Methods

### Preparation and Equipment.

The precursor solution for all experiments was 250 mM acetylene dicarboxylic acid monopotassium salt, 250 mM sodium sulfite, and 7 mM ruthenium catalyst [RuCp*(CH_3_CN)_3_]PF_6_ in D_2_O, which was prepared by dissolving the solids with heating and sonication. The solution additionally contained 250 mM NaOD, to be equimolar with the starting material. The sodium sulfite is added to increase the rate of reaction (20). Oxygen was removed from the solution by bubbling nitrogen through for 5 min. All chemicals were purchased from Sigma-Aldrich.

All NMR experiments (unless otherwise stated) were performed in a 1.4-T ^1^H-^13^C dual-resonance SpinSolve NMR system (Magritek).

Parahydrogen at >99% enrichment was generated by passing hydrogen gas (>99.999% purity) over a hydrated iron-oxide catalyst in a cryostat operating at 25 K (Advanced Research Systems).

For the magnetic field sweep, an MS-2 magnetic shield (Twinleaf LLC) was used to provide a 10^5^ shielding factor against external magnetic fields. No static shim fields were required, since the residual field within the shield was on the order of 1 nT. The time-dependent applied magnetic field was generated using the built-in B_y_ shim coil, with current supplied by a Kea2 spectrometer with 1-µs time precision.

A home-built Halbach magnet array was used to provide a 100-mT static magnetic field over a cylindrical region in space of 22 mm radius and 150 mm length in which the precipitation and redissolution procedure was carried out. This was achieved by arranging two rings of eight 0.5 × 0.5 × 2 in^3^ neodymium N52 magnets (1.4 T remanence) in a cylindrical Halbach dipole array, with the magnet centers lying on a circle of radius 45 mm.

The reactor was constructed of stainless steel with an internal volume of 20 mL, as shown in Fig. 1. Polyether ether ketone (PEEK) tubing of 1/8 in. outer diameter (O.D.) was used to flow the *para*-enriched hydrogen gas, and 1/16-in. O.D. 0.5-mm inner diameter polytetrafluoroethylene (PTFE) capillaries were used for all solution flow. These tubes were connected to the reactor via 1/4–28 PEEK fittings (part numbers P-249 and P-349, IDEX LLC), and to the gas flow-control manifold via Swagelok fittings (Swagelok). The reactor was wrapped in two heater mats (part number 798–3753, RS Components), and these were connected to a power supply, with the current set to heat the reactor to 85 °C.

### Hydrogenation and Field Sweep.

For all hydrogenation experiments, a 2 mL aliquot of precursor sample was loaded into the reactor via syringe injection, and given 10 s to reach 85 °C. The reactor was sealed, and parahydrogen was bubbled into the reaction solution at ∼6 L/min for a time (60 s unless otherwise specified) at a pressure of 8.5 bar. The flow rate was set by a needle valve at the outlet of the gas manifold. After bubbling, the sample was pneumatically ejected through a PTFE capillary into a 10-mm NMR tube in the magnetic shield underneath by manually opening a two-way microfluidic valve. To prevent the sample from passing through any fields that could lead to undesired state mixing during sample transport in/out of the shield, a penetrating solenoid was used to provide a 10-µT guiding field. Upon landing in the 10-mm NMR tube, the field was nonadiabatically (rapidly) dropped to 50 nT and then adiabatically increased to 1 µT in 2 s to transfer the proton singlet order into ^13^C magnetization. The control sample was 0.6 mL of the solution extracted through a 1/16-in. PTFE capillary into a syringe.

### Purification Procedure.

In experiments involving precipitation/redissolution, 1.25 mL of the sample in the 10-mm NMR tube was extracted into a syringe containing 0.75 mL of 1 M sodium fumarate in D_2_O. The remaining 150 µL of reaction solution was lost as droplets in the transfer capillaries and containers. The 2 mL sample was then rapidly injected into 1 mL of 12 M HCl at room temperature on a glass sinter (grade 4) atop a vacuum flask. Fumaric acid crystals immediately precipitated out as a white solid. The residual solution was removed by vacuum filtration, and the fumaric acid crystals were redissolved in 1 mL of 3 M NaOD to produce the purified sample.

### Enzyme Experiments.

For the experiment to observe enzymatic conversion to malate, after the purification procedure, 450 µL of the purified sample was injected into a 5-mm NMR tube containing 10 µL of fumarase enzyme in 150 µL of pH 7 phosphate buffer solution. The NMR tube was immediately inserted into the benchtop magnet for ^13^C signal acquisition using 15° flip-angle pulses every 7 s to monitor the hyperpolarized NMR signals over time.

## Supplementary Material

Supplementary File

## Data Availability

All study data are included in the article and/or *SI Appendix*.

## Footnotes

*LD_50_ is the median lethal dose. IC_50_ is the half-maximal inhibitory concentration.
