# Supplementary material for: Rapid hyperpolarization and purification of the metabolite fumarate in aqueous solution
Source: Proc Natl Acad Sci U S A. 2021 Mar 22;118(13):e2025383118. doi: 10.1073/pnas.2025383118 (PMC8020773; doi:10.1073/pnas.2025383118)
Supplement: Supplementary File [file pnas.2025383118.sapp.pdf]

Rapid hyperpolarization and purification of the metabolite fumarate in aqueous solution – Supporting Information

Stephan Knecht[a,†], John W. Blanchard[b,†], Danila Barskiy[c], Eleonora Cavallari[d], Laurynas Dagys[e], Erik Van Dyke[c], Maksim Tsukanov[c], Bea Bliemel[c], Kerstin Münnemann[f], Silvio Aime[d], Francesca Reineri[d], Malcolm H. Levitt[e], Gerd Buntkowsky[a], Alexander Pines[c], Peter Blümler[g], Dmitry Budker[b,g], James Eills[b,g,*]

a) Eduard-Zintl-Institute for Inorganic Chemistry and Physical Chemistry, Technical University Darmstadt, 64287 Darmstadt, Germany
b) Helmholtz-Institut Mainz, GSI Helmholtzzentrum für Schwerionenforschung GmbH, 55128 Mainz, Germany
c) Department of Chemistry, University of California, Berkeley, U.S.A.
d) Dept. of Molecular Biotechnology and Health Sciences, University of Torino, Torino, Italy
e) University of Southampton, Southampton, United Kingdom
f) Technical University of Kaiserslautern, Kaiserslautern, Germany
g) Johannes Gutenberg University, D-55090 Mainz, Germany
(†) These authors contributed equally to this work
(*) Correspondence: eills@uni-mainz.de

Effective relaxation induced by pulsing

In experiments to measure the fumarate ^{13}C T_1 times, 10° flip-angle pulses were applied every 5 s to excite the NMR signals for acquisition. This itself induces effective relaxation of the hyperpolarized magnetization, and the time constant for this process, T_P, is given by:

$$T_\mathrm{P} = \frac{\delta}{\log(\cos(\theta))},$$

where θ is the flip angle used, and δ is the time between pulses. In these experiments T_P was calculated to be 327 s.

Sample purification analysis

To prepare a control sample, 2 mL of precursor solution was hydrogenated for 60 s, and 0.6 mL of the reacted solution was extracted for analysis without further modification. To prepare a purified sample, 1.25 mL of the reacted solution was extracted into a syringe containing 0.75 mL of 1 M sodium fumarate in D_2O. This was added to 1 mL 12 M HCl to precipitate out solid fumarate, and the residual solution was vacuum filtered off. The solid was washed twice with H_2O and twice with acetone. The dry solid was then dissolved in 1 mL 3 M NaOD, and 0.8 mL was extracted for analysis.

To compare the purity of these two solutions, ^{1}H NMR spectra were acquired, and are shown in Fig. S1. The ^{1}H NMR spectra were acquired at 11.7 T using 30° flip angle pulses, using 256 transients with a 30 s pre-scan delay. A peak corresponding to the olefinic protons in fumarate is clearly visible at 6.6 ppm, and peaks corresponding to water protons are visible from 4.7-5.1 ppm, with the shift caused by the difference in pH between the two solutions.

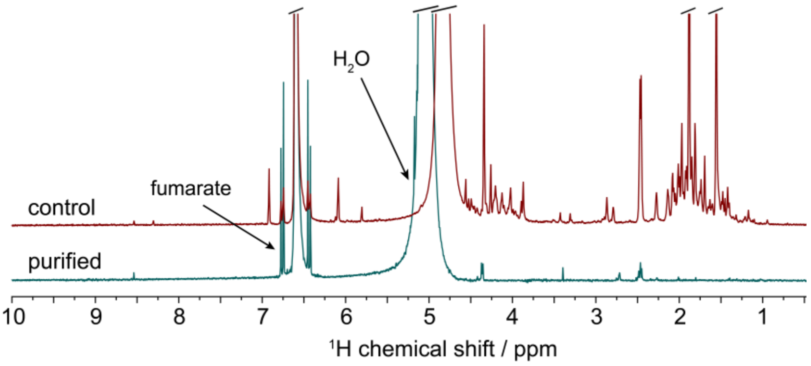

Fig. S1: ^{1}H NMR spectra of a purified sample and a control sample. Unlabelled peaks correspond to impurities. The water peak shift is caused by a pH difference between the solutions.

High-performance liquid chromatography (HPLC) analysis was also used to study the two samples, and the results are shown in Fig. S2. 800 µL of the purified solution was freeze dried to yield 140 mg of white powder. 1 mg of the powder was dissolved in water and diluted with an acetonitrile/water mixture (50/50 v/v). As a comparison, a 10 mM solution of the starting material (ADC) and a 10 mM solution of fumarate in water were prepared and diluted by the same ratio. The samples were analyzed by reverse phase HPLC. In the purified sample, the peak at 2.64 min retention time was assigned to the ADC educt. A comparison of the integrals between the sample and the reference yields a concentration of 1.8 mM ADC in the purified sample, although there is significant error on this value.

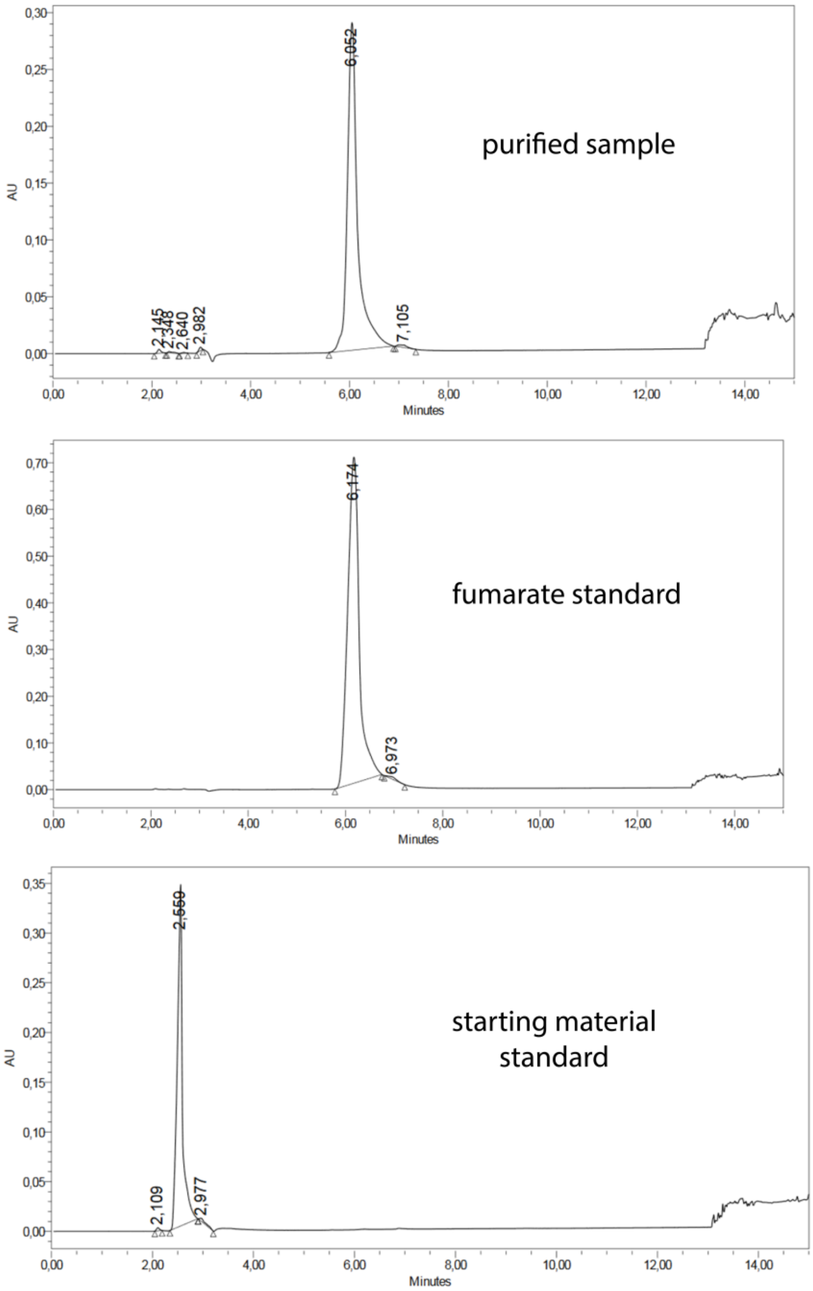

Fig. S2: HPLC data of the purified sample (top), fumarate standard (middle), and starting material standard (bottom).
